# AI-augmented reliability in CI/CD: a framework for predictive, adaptive, and self-correcting pipelines

**DOI:** 10.3389/frai.2026.1776546

**Published:** 2026-04-01

**Authors:** Rohit Dhawan, Mohit Dhawan

**Affiliations:** Independent Researcher, Edmonds, WA, United States

**Keywords:** adaptive systems, artificial intelligence, CI/CD, DevOps, machine learning, pipeline automation, predictive analytics, software reliability

## Abstract

Modern CI/CD pipelines face a critical challenge. While AI tools accelerate code generation, static pipelines have become the primary bottleneck to delivery velocity. Flaky tests and pipeline noise create a persistent challenge, with reported failure rates ranging from 11 to 27 percent for test flakiness and 5–16 percent for noise-induced build failures. This forces teams to spend more time investigating false failures than building features. As systems scale across regions and dependencies, these problems compound and threaten the fundamental promise of continuous delivery. We introduce a framework that transforms CI/CD pipelines from deterministic scripts into intelligent, adaptive systems. At its core is the Sense-Analyze-Predict-Act-Learn loop, which we call SAPAL. This loop extends classical adaptive models with CI/CD specific capabilities including flakiness characterization, dependency risk scoring, multi-region awareness, and developer feedback. We operationalize this loop through a five-layer architecture spanning data collection, reliability intelligence, predictive modeling, adaptive execution, and human-AI collaboration. Three novel metrics quantify pipeline intelligence. Pipeline Health Index measures overall reliability. Test Stability Score identifies flaky patterns. Failure Prediction Confidence validates model accuracy. Three scenarios demonstrate application to real CI/CD challenges. Intelligent retry strategies, grounded in empirical studies of flaky test detection and resolution, project 60 percent reduction in flaky-induced build failures. ML-based test selection techniques from recent literature suggest 50 to 80 percent reduction in feedback time. Stability-aware deployment orchestration adapts rollout strategies to regional reliability patterns. These projections synthesize findings from published component studies rather than measurements from unified framework deployment. By enabling pipelines to learn from executions, predict with calibrated confidence, and adapt to behavior patterns, this framework provides a practical path toward reliable delivery at scale where intelligence is essential, not optional.

## 1 Introduction

Software delivery pipelines have become essential infrastructure for modern engineering organizations. Industry surveys show that large technology companies routinely deploy hundreds of microservices across multiple regions. Each service evolves daily in response to customer needs, dependency changes, and infrastructure updates (Forsgren et al., 2018; CD Foundation, 2024). Despite this operational complexity, most CI/CD pipelines still operate as fixed, rule-based systems. They execute the same tests, follow identical workflow steps, and apply uniform decision logic. This happens regardless of actual system conditions, dependency state, or environmental context at execution time.

This fundamental mismatch between static pipeline behavior and dynamic system reality creates significant reliability and productivity challenges. Teams routinely encounter recurring build failures, unstable integration tests, noisy alerts, and deployments that fail for reasons unrelated to code changes (Luo et al., 2014; Bell et al., 2018). Research shows that many failures stem not from genuine defects but from pipeline noise. Flaky tests fail intermittently due to timing issues or environment instability, affecting 11–27 percent of tests in large industrial projects and accounting for 5–16 percent of build failures (Lam et al., 2019; Luo et al., 2014). Test environments become unstable when shared resources are overloaded. Dependencies break when upstream systems change without warning. Configuration drift causes tests to succeed in some regions but fail in others. As systems grow in scale and distribution, these issues compound. The result is bottlenecks that slow delivery velocity while increasing operational risk (Leite et al., 2019).

At the same time, engineering organizations collect extensive telemetry from previous failures, production rollbacks, dependency updates, system load patterns, regional differences, and test execution history. Today's pipelines do not leverage this information systematically. They exhibit no memory and demonstrate no learning behavior across executions. A pipeline that fails today for a known reason will fail again next week for the same reason. This forces manual intervention and consumes engineering time that could be invested in feature development. The result is a CI/CD system that appears busy but behaves blindly. It cannot distinguish signal from noise or adapt its behavior based on accumulated evidence.

### 1.1 Motivation

Modern services operate across many regions, depend on dozens of upstream and downstream systems, and function under constantly changing load patterns (Zhang et al., 2018). Consider a representative scenario. A microservice is deployed to seven regions. Each region may exhibit different operational characteristics. One region operates on older infrastructure with slower response times. Another region is undergoing a database migration. A third region regularly experiences noisy integration tests due to high customer traffic during business hours. Despite these documented differences, conventional pipelines treat every region identically. They trigger the same canary checks, execute the same test suites, and apply identical rollout strategies everywhere.

This uniform approach leads to predictable problems. False failures occur in stable regions and waste engineering time. Real issues are missed in unstable regions and escape to production. Unnecessary test reruns delay feedback. Deployments are blocked by transient failures. Avoidable customer facing incidents occur.

AI-augmented CI/CD addresses these challenges by enabling pipelines to learn from real system conditions and adapt their decisions to reflect actual behavior patterns. This enables several capabilities. Pipelines can execute only the most relevant tests for a given change. They can apply stricter validation checks to high-risk code paths or dependencies. They can implement intelligent retry logic based on failure signatures and test stability history. They can adjust rollout strategies based on regional stability characteristics (Pan et al., 2021). The pipeline develops institutional memory and becomes progressively more effective at avoiding known failure modes while adapting to emerging patterns.

The importance of such adaptive systems increases as organizations embrace multi-region architectures, expand microservice ecosystems, and accelerate toward continuous delivery (Forsgren et al., 2018). Larger systems generate more noise. More dependencies introduce more failure modes. More regions create more behavioral variations. Greater deployment frequency amplifies the cost of false positives and reduces tolerance for manual intervention. AI-augmented reliability offers a structured path toward safer, faster, and more predictable software delivery in environments where complexity is rising faster than teams can scale.

### 1.2 Research gap

Existing research demonstrates that machine learning techniques can improve specific aspects of CI/CD workflows. Studies document applications in test case prioritization (Spieker et al., 2017; Pan et al., 2021), failure prediction (Bertolino et al., 2020; Saidani et al., 2022), and flaky test detection (Lam et al., 2019; Pinto et al., 2020). Industry tools such as Launchable, Harness, and DataDog apply ML for test selection, deployment verification, and anomaly detection respectively. However, these contributions address isolated problems without providing a unified conceptual framework for intelligent, adaptive CI/CD systems.

Current approaches exhibit several fundamental gaps. First, they focus on individual pipeline stages rather than integrating capabilities across the entire delivery workflow. Test prioritization improvements do not connect to deployment risk assessment. Failure prediction does not inform retry strategies. Flaky test detection operates independently from environment routing decisions. Second, existing techniques react to failures after they occur rather than predicting and preventing them proactively based on learned patterns. Third, proposed solutions lack explicit learning mechanisms that enable continuous improvement across thousands of pipeline executions. Fourth, there exists no comprehensive set of metrics for evaluating whether a pipeline is learning effectively, making accurate predictions, or improving its decisions over time.

Most critically, no existing work unifies test intelligence, failure prediction, dependency risk modeling, regional deployment variability, and adaptive execution within a single conceptual framework that explains how these capabilities should work together to create end-to-end pipeline reliability. The MAPE-K model, which stands for Monitor, Analyze, Plan, Execute, and Knowledge, provides a reference architecture for self-adaptive systems (Kephart and Chess, 2003). However, it was designed for long-running services with stable execution environments. CI/CD pipelines present fundamentally different characteristics. Executions are short-lived and measured in minutes. Triggers are commit-driven rather than continuous. Cross-stage causal dependencies exist where build leads to test leads to deploy. Learning must occur between runs rather than within runs. These differences necessitate a domain-specific adaptation of adaptive system principles for software delivery contexts.

### 1.3 Contributions

This paper makes four primary contributions to the field of intelligent software delivery.

First, we propose the Sense-Analyze-Predict-Act-Learn loop, which we call SAPAL. This is a conceptual model that describes how CI/CD pipelines can become intelligent, adaptive systems. The SAPAL loop extends classical adaptive frameworks such as MAPE-K by incorporating CI/CD specific constructs. These include multi-region deployment awareness, dependency risk quantification, flakiness characterization, and developer feedback integration. We explicitly compare SAPAL with MAPE-K and demonstrate why existing adaptive system models require modification for software delivery contexts. The loop consists of five stages that transform deterministic pipelines into learning systems. These stages are sensing real-world conditions from code changes through production behavior, analyzing patterns to identify risks and anomalies, predicting outcomes including test failures and deployment risks, acting to adapt execution strategies based on predictions and confidence levels, and learning from results to improve future decisions.

Second, we introduce a five-layer architectural framework that operationalizes the SAPAL loop through concrete components and clear interfaces. The architecture defines distinct responsibilities for data collection and observability, reliability intelligence and pattern recognition, predictive modeling and forecasting, adaptive execution and decision making, and human-AI collaboration and feedback. Each layer addresses specific technical challenges while maintaining modularity that supports incremental adoption. The layered design provides a practical blueprint for organizations seeking to build intelligent CI/CD systems without requiring wholesale replacement of existing infrastructure.

Third, we propose three core reliability metrics designed specifically for adaptive pipelines. Pipeline Health Index measures overall system reliability by combining multiple signals. Test Stability Score quantifies test consistency to identify flaky behavior. Failure Prediction Confidence evaluates the accuracy and calibration of predictive models. We provide theoretical grounding for each metric formulation, discuss calibration strategies for organizational contexts, and explain how these metrics shift focus from raw speed to meaningful reliability and continuous improvement. Unlike traditional CI/CD metrics that measure throughput and cycle time, these metrics quantify decision quality, prediction accuracy, and learning effectiveness.

Fourth, we demonstrate framework applicability through three detailed application scenarios grounded in patterns and parameters from published research. The scenarios address flaky test management using intelligent retry and quarantine strategies, selective test execution through risk-based prioritization, and regional deployment using stability-aware rollout orchestration. We clearly distinguish these literature-grounded demonstrations from empirical validation while projecting expected outcomes based on results reported in prior studies. These scenarios illustrate how the SAPAL loop and five-layer architecture combine to address real CI/CD reliability challenges.

To the best of our knowledge, this work represents the first comprehensive framework that integrates predictive analytics, adaptive decision-making, and continuous learning for CI/CD reliability within a unified conceptual model grounded in adaptive systems theory but adapted for software delivery contexts. We present SAPAL as a theory-informed framework that generates testable predictions about CI/CD reliability outcomes. While this work provides theoretical foundations and architectural blueprints without empirical validation, it follows the precedent of foundational models like MAPE-K in autonomic computing, establishing a reference architecture upon which future hypothesis-driven empirical research can build.

#### 1.3.1 Contribution taxonomy

This framework makes two distinct types of contributions. The ML/AI contribution lies in formalizing prediction as a first-class control stage within CI/CD governance (the SAPAL loop), defining reliability-aware metrics (PHI, TSS, FPC) that quantify learning effectiveness, and establishing calibration-aware decision policies that couple predictive confidence with operational risk. The systems contribution provides a layered architectural blueprint for integrating these ML capabilities into production pipelines, along with concrete adaptive execution strategies and safety-constrained decision logic. The primary novelty lies in adapting predictive control principles to the unique constraints of short-lived, commit-driven pipeline executions, where decisions must be made rapidly under uncertainty while preserving correctness.

### 1.4 Paper organization

The remainder of this paper proceeds as follows. Section 2 provides essential background on CI/CD evolution, current reliability challenges, AI applications in software engineering, and the relationship between SAPAL and classical adaptive system models. Section 3 presents the complete AI-augmented reliability framework including the SAPAL loop, five-layer architecture, reliability metrics, and decision logic. Section 4 demonstrates framework application through three scenarios addressing flaky tests, selective execution, and regional deployment. Section 5 discusses practical implications, adoption strategies, and limitations. Section 6 concludes with a summary of contributions and directions for future research including empirical validation requirements.

Table 1 provides an overview of the framework's major components and their roles in creating intelligent CI/CD systems.

**Table 1:** Overview of framework components and contributions.

Component	Description	Key benefit
SAPAL loop	Sense, Analyze, predict, act, learn cycle	Enables continuous improvement through feedback
Five-layer architecture	Data, intelligence, prediction, execution, collaboration	Provides modular implementation blueprint
Core metrics	PHI, TSS, FPC	Quantifies intelligence and reliability
Application scenarios	Flaky tests, selective execution, regional deployment	Demonstrates practical feasibility

## 2 Background

This section provides essential context for understanding the AI-augmented reliability framework. We begin by examining the evolution of CI/CD pipelines and the challenges they face at scale. We then review how AI techniques are being applied to software engineering problems. Finally, we discuss adaptive system models and explain why classical frameworks require modification for CI/CD contexts.

### 2.1 CI/CD evolution and current challenges

Continuous integration and continuous delivery have become standard practice for modern engineering teams. Early CI/CD systems automated repeatable tasks such as compiling code, executing tests, and deploying to environments (Duvall et al., 2007; Humble and Farley, 2010). This approach worked well for monolithic systems where teams ran comprehensive test suites and followed fixed deployment procedures.

As organizations adopted microservice architectures (Newman, 2015), CI/CD pipelines expanded dramatically. Modern pipelines manage thousands of tests, region-specific configurations, complex dependencies, deployment gates, and canary releases (Bass et al., 2015). Enterprise pipelines now coordinate rollouts across multiple regions and services owned by different teams.

As described in Section 1, most pipelines continue to operate as static, rule-based systems despite these architectural changes. The DevOps community has acknowledged persistent problems including flaky tests, environment instability, dependency drift, and pipeline noise (Leite et al., 2019). Current approaches to these problems rely on manual intervention, reruns, retries, test quarantines, and additional approval gates. These solutions slow delivery without improving underlying reliability.

Research has documented the widespread impact of these challenges on software quality and developer productivity. Luo et al. (2014) conducted an empirical analysis showing that flaky tests account for a significant portion of test failures in practice. Lam et al. (2019) documented widespread flaky test problems in large industrial projects. Thorve et al. (2018) reported even higher rates in certain Android projects, with flaky test rates reaching 45 percent. Teams spend considerable time diagnosing and addressing these issues, often without systematic approaches or tools that can distinguish flaky failures from genuine defects.

Environment instability represents another major challenge. Hilton et al. (2017) found that infrastructure issues are a significant source of CI failures, with resource contention and environment instability causing spurious test failures. Tests that pass in isolated environments may fail when executed on shared infrastructure under load. This variability makes it difficult for teams to trust test results or maintain consistent quality standards.

Dependency management at scale introduces additional complexity. Modern software depends on numerous external libraries and internal services that evolve independently (Cox, 2019). When dependencies update, changes can introduce subtle breaking changes that are difficult to detect through standard testing approaches. Teams lack systematic ways to assess dependency risk or prioritize validation efforts based on the likelihood and impact of breaking changes.

### 2.2 AI applications in software engineering

Machine learning and artificial intelligence techniques are increasingly applied to software engineering tasks. Recent surveys document applications in defect prediction, code generation, program repair, test generation, code review automation, effort estimation, and software analytics (Zhang et al., 2013; Watson et al., 2022). These studies demonstrate that data-driven approaches can augment developer capabilities and improve quality outcomes when applied appropriately.

Several areas have shown particular promise for CI/CD contexts. Test case prioritization uses ML models to predict which tests are most likely to fail for a given code change (Pan et al., 2021). Researchers have applied techniques including random forests, neural networks, and genetic algorithms to improve test selection efficiency. Spieker et al. (2017) introduced RETECS, a reinforcement learning approach for test prioritization in continuous integration. Their evaluation on industrial case studies showed that adaptive prioritization can reduce test execution time while maintaining fault detection effectiveness. Zhao et al. (2024) demonstrated that pretraining prioritization models with cross-project data followed by fine-tuning with project-specific data achieves state-of-the-art performance, with optimal test orderings on 80 percent of evaluated subjects.

Failure prediction represents another active research area. Saidani et al. (2022) developed DL-CIBuild, a deep learning approach using LSTM recurrent neural networks to predict CI build outcomes. Their evaluation on over 91,000 CI builds from 10 projects showed that LSTM-based models outperform traditional machine learning approaches for both cross-project and online prediction scenarios. Hassan and Wang (2017) introduced change-aware build prediction models that achieve 75 percent accuracy in predicting build outcomes on large-scale industrial projects.

Flaky test detection has received considerable attention given its practical importance. Bell et al. (2018) developed DeFlaker, which identifies flaky tests with 96 percent precision and 61 percent recall. Parry et al. (2023) introduced CANNIER, combining test rerunning with machine learning models to detect flaky tests more efficiently. Their approach reduces detection time by an order of magnitude compared to pure rerunning while maintaining better accuracy than ML alone.

Industry tools demonstrate practical applications of these techniques. Launchable applies predictive test selection to reduce CI execution time by learning which tests are most likely to fail based on code change characteristics. Harness automates deployment verification using ML-driven canary analysis, comparing metrics between new and baseline deployments to detect regressions before full rollout. DataDog uses machine learning for anomaly detection in application metrics, automatically identifying unusual patterns in time-series data and correlating them with deployment events.

However, the use of AI inside CI/CD pipelines themselves remains limited to isolated improvements. Existing research focuses on single-step enhancements such as predicting failing tests, selecting test subsets, or identifying flaky behavior. These contributions provide value but do not define an end-to-end framework for intelligent, adaptive CI/CD systems that integrate multiple capabilities across the entire delivery workflow.

### 2.3 Adaptive systems and the MAPE-K model

Self-adaptive systems modify their behavior in response to changes in their operating environment or internal state. Salehie and Tahvildari (2009) surveyed self-adaptive software systems and identified key challenges including when to adapt, what to adapt, and how to ensure adaptation correctness. These systems follow feedback loops consisting of monitoring, analysis, planning, and execution stages.

The MAPE-K model, introduced by Kephart and Chess (2003) as part of the autonomic computing vision, provides a widely cited reference architecture for self-adaptive systems. The model consists of four main stages and a knowledge component. The Monitor stage collects data about the system and its environment. The Analyze stage processes this data to understand current state and identify problems. The Plan stage determines what actions should be taken to address identified issues. The Execute stage carries out these actions. The Knowledge component maintains information about the system, its environment, and adaptation policies.

MAPE-K has influenced research and practice in domains including cloud computing, robotics, and network management. However, it was designed for long-running systems with stable execution environments. Applications of MAPE-K typically assume continuous operation where the system can monitor its state over extended periods, adapt its behavior within a single execution context, and maintain persistent knowledge across its operational lifetime.

CI/CD pipelines present fundamentally different characteristics that make direct application of MAPE-K problematic. First, pipeline executions are short-lived processes measured in minutes rather than hours or days. Each execution is triggered by a code commit and runs to completion independently. Second, pipelines follow strict stage ordering where build must complete before test, and test must complete before deploy. This creates cross-stage causal dependencies that do not exist in typical autonomic systems. Third, each pipeline execution operates in isolation with no persistent state. Learning must occur between executions rather than within a single long-running process. Fourth, pipelines must make rapid decisions under time pressure, typically producing results within 10–30 min to maintain developer productivity.

These differences necessitate adaptations to the classical MAPE-K model for CI/CD contexts. The framework we propose in this paper, centered on the SAPAL loop, addresses these CI/CD-specific requirements while preserving the core principle of feedback-driven adaptation.

SAPAL diverges from MAPE-K in five important ways. First, SAPAL introduces prediction as an explicit first-class stage. CI/CD pipelines operate in short cycles where the system must decide quickly whether to run tests, skip tests, change environments, or slow down a rollout. These decisions require probability estimates and confidence scoring. Classical MAPE-K does not include probabilistic forecasting or model calibration. Separating prediction from action changes the sequence and timing of adaptation and creates a reliability loop that reflects the realities of CI/CD systems.

Second, SAPAL models multi-stage causality explicitly. Pipelines have a strict order of stages such as build, test, deploy, and monitor. Issues at one stage influence actions in subsequent stages. SAPAL performs causal reasoning across these stages. MAPE-K treats the system as a single state that is monitored and adapted without explicit stage boundaries.

Third, SAPAL addresses the short-lived nature of CI/CD executions. Each pipeline run is independent from the next and lasts only a few minutes. Adaptation must occur inside each run, and models must be updated across runs. MAPE-K was designed for continuously running systems with stable execution environments, so it does not address this temporal constraint.

Fourth, SAPAL focuses on continuous learning rather than static knowledge. CI/CD pipelines experience drift in many forms. Test flakiness patterns change. Dependencies evolve. Regional stability shifts. Model accuracy degrades. SAPAL retrains models based on feedback from each pipeline execution. This makes learning an active and ongoing process rather than a passive knowledge store as in MAPE-K.

Fifth, SAPAL incorporates explicit human involvement. Pipelines require developer trust for actions such as selective testing, skipping tests based on prediction, or slowing deployments in risky regions. SAPAL includes explanation, override, and feedback channels that allow developers to influence the behavior of the adaptive system. MAPE-K assumes full automation and does not model human collaboration.

Table 2 summarizes the key differences between MAPE-K and SAPAL, highlighting how our framework adapts classical adaptive system principles for software delivery contexts.

**Table 2:** Comparison of SAPAL loop and MAPE-K model.

Aspect	MAPE-K	SAPAL
Domain	Long-running autonomic systems	Short-lived, commit-driven CI/CD executions
Sense/monitor	System events and metrics	Code changes, test flakiness, dependency drift, region behavior
Analyze	Pattern identification	Multi-stage causal analysis across build, test, deploy
Plan	Rule-based planning	Separate prediction stage with probabilistic forecasting
Predict	Not present	Explicit stage for failure prediction, risk scoring
Act/execute	Generic actuation	Test selection, retry decisions, regional rollouts
Learn/Knowledge	Static knowledge base	Continuous model retraining, drift handling, feedback
Human role	Limited and implicit	Explicit collaboration, overrides, explanations

The next section presents the complete AI-augmented reliability framework, beginning with detailed explanation of the SAPAL loop stages and their relationship to pipeline intelligence.

## 3 The AI-augmented reliability framework

This section presents the complete framework for AI-augmented CI/CD reliability. We begin by introducing the SAPAL loop and explaining how it extends classical adaptive models for CI/CD contexts. We then describe a five-layer architecture that operationalizes this loop through concrete components. Next, we define three core metrics for measuring pipeline intelligence and reliability. Finally, we present the decision logic that guides adaptive pipeline behavior.

### 3.1 The SAPAL loop

**Figure 1:**
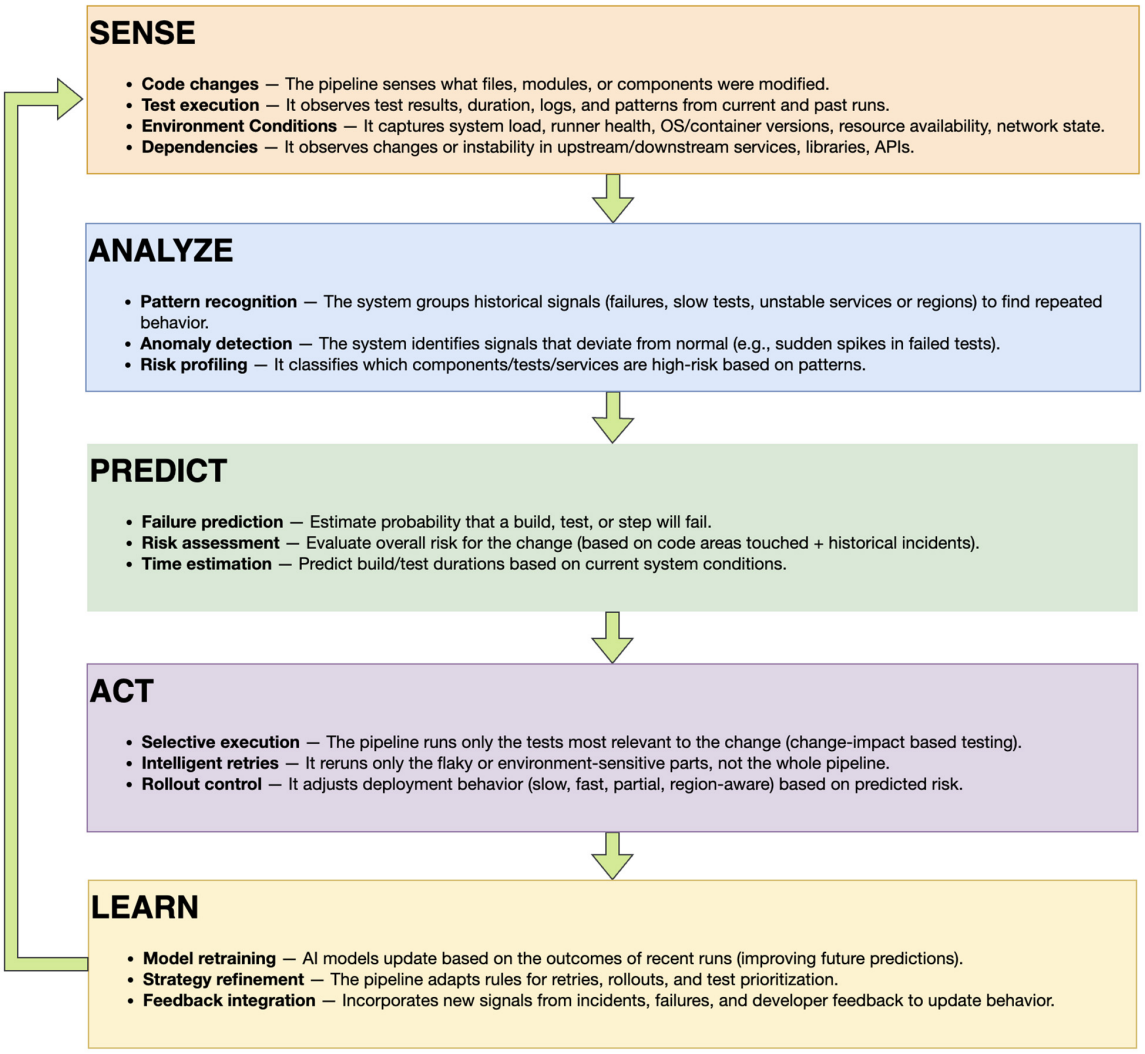
The Sense-Analyze-Predict-Act-Learn (SAPAL) Loop for AI-Augmented CI/CD Reliability. The loop operates continuously across pipeline executions, sensing system conditions, analyzing patterns, predicting outcomes, adapting execution, and learning from results.

The Sense-Analyze-Predict-Act-Learn loop provides a conceptual model for transforming static CI/CD pipelines into intelligent, adaptive systems. The loop operates continuously throughout the software delivery lifecycle. It collects signals from code changes, test executions, deployments, and production behavior. It analyzes patterns and correlations in this data. It predicts risks and likely outcomes. It adapts execution strategies based on predictions. It learns from results to improve future decisions. Figure 1 illustrates the structure and flow of this loop.

#### 3.1.1 Stage 1: Sense

The first stage collects real-time and historical data from across the software delivery system. Unlike traditional pipelines that only track pass or fail status, the sensing stage captures rich contextual information reflecting actual system conditions.

The pipeline senses code changes including files modified, modules affected, and change frequency. It captures test execution data including pass or fail status, execution time, error messages, flakiness patterns, and retry history. Build and deployment metrics include build duration, dependency versions, deployment region, and rollback events.

Environment conditions such as infrastructure load, network latency, and cache hit rates enable pipelines to distinguish real failures from infrastructure noise. Dependency state tracking covers service versions, API schema changes, and breaking change alerts. Production signals including error rates, latency distributions, and regional performance differences inform pipeline decisions about rollout strategies.

The sensing stage leverages existing observability infrastructure and standardized telemetry formats, capturing signals that matter for reliability decisions.

#### 3.1.2 Stage 2: Analyze

The analysis stage processes sensed data to identify patterns, correlations, and anomalies, transforming raw signals into actionable insights about system behavior and reliability risks.

Pattern recognition identifies recurring failure patterns, common error signatures, and temporal correlations (Han et al., 2011). For example, the pipeline may discover that a specific test fails when a dependent service undergoes maintenance. Anomaly detection flags deviations including unusual test durations, unexpected dependency changes, or regional performance degradation (Chandola et al., 2009).

Root cause correlation links failures with potential causes such as code changes, environment shifts, or infrastructure events, distinguishing code defects from external failures. Flakiness characterization identifies tests that fail intermittently and determines whether failures are timing dependent, environment dependent, or order dependent (Lam et al., 2019).

Risk profiling assesses risk factors for changes, regions, and deployments. Dependency impact analysis traces how changes propagate through the system (Cox, 2019).

#### 3.1.3 Stage 3: Predict

The prediction stage uses analyzed data to forecast future outcomes. This enables proactive decision making rather than reactive responses. Predictions are probabilistic and include confidence estimates.

Failure prediction forecasts which tests are likely to fail given the current code change, environment state, and dependency configuration (Bertolino et al., 2020). Predictions guide test selection and prioritization. High-confidence predictions allow the pipeline to skip stable tests or focus resources on risky areas. Flakiness prediction estimates whether a test is likely to exhibit flaky behavior under current conditions (Pinto et al., 2020).

Build time prediction estimates how long a build will take based on code changes, test selection, and infrastructure load (Hassan and Wang, 2017). Deployment risk assessment predicts the likelihood of rollback, error rate increases, or performance degradation before deploying to production (Vassallo et al., 2019). Regional behavior prediction forecasts how a deployment will perform in different regions based on regional history, infrastructure differences, and traffic patterns.

Prediction models use supervised learning techniques trained on historical data (Pan et al., 2021). Models are continuously updated as new data arrives. Predictions include confidence scores that reflect model certainty. Low-confidence predictions trigger conservative strategies or human review.

#### 3.1.4 Stage 4: Act

The action stage uses predictions to adapt pipeline behavior. This is where intelligence translates into concrete decisions that improve reliability and efficiency.

Selective test execution runs only tests likely to fail or tests affected by code changes based on failure predictions (Yaraghi et al., 2023). This reduces feedback time without sacrificing coverage. Intelligent retries decide whether to retry based on flakiness predictions and failure signatures (Bell et al., 2018). If the failure matches a known flaky pattern, the pipeline retries automatically. If the failure appears genuine, it fails fast and alerts developers immediately.

Adaptive timeouts adjust dynamically based on environment conditions and historical execution times. Environment routing assigns tests to environments that minimize flakiness and maximize reliability (Hilton et al., 2017). Risk-based rollout adapts deployment strategies to predicted risk, with high-risk changes deploying to small canaries with strict monitoring (Vassallo et al., 2019). Dynamic gating applies stricter quality gates to high-risk changes and relaxes gates for low-risk changes.

Actions are reversible and auditable. The pipeline logs every decision, its rationale, and its outcome. This transparency builds trust and enables continuous improvement.

#### 3.1.5 Stage 5: Learn

The learning stage closes the loop by updating models and strategies based on observed outcomes. This ensures the pipeline improves over time rather than repeating mistakes.

Model retraining updates prediction models periodically using the latest data (Spieker et al., 2017). When new failure patterns emerge or system behavior changes, models adapt. Strategy refinement evaluates the effectiveness of actions and refines decision policies based on outcomes. Developer feedback is integrated into learning, with repeated overrides indicating model errors or missing context.

When incidents occur despite predictions, the pipeline analyzes what went wrong through failure post-mortems, generating new training data and policy updates. Continuous evaluation monitors pipeline performance using metrics such as prediction accuracy, false positive rate, time saved, and developer satisfaction (Forsgren et al., 2018). Learned patterns and strategies are shared across teams and projects through knowledge sharing mechanisms.

Learning is gradual and conservative. The pipeline does not make abrupt changes based on single events. It accumulates evidence and adjusts policies incrementally, preventing overreaction to outliers while ensuring continuous improvement.

#### 3.1.6 Loop integration

The SAPAL loop operates continuously and iteratively. Each pipeline execution contributes data to sensing, which informs analysis, which improves predictions, which guide actions, which generate outcomes that feed learning. Over time, the loop accumulates knowledge about the specific characteristics of the codebase, team, infrastructure, and deployment environment.

The loop operates at multiple time scales. Real-time sensing and prediction occur during individual builds. Analysis and learning happen in the background, processing aggregated data from many executions. The loop is modular and extensible, with new sensors, prediction models, and actions integrated independently. Importantly, the loop includes humans. Developers provide feedback, override decisions, and contribute domain knowledge. Operators monitor loop behavior and intervene when necessary. The loop augments human intelligence rather than replacing it.

#### 3.1.7 What SAPAL enables that static pipelines cannot

The SAPAL loop transforms CI/CD from reactive automation into proactive intelligence. Static pipelines execute the same steps regardless of context and repeat the same failures indefinitely. SAPAL-augmented pipelines sense actual system conditions, predict likely outcomes with calibrated confidence, adapt execution strategies to match predicted risk, and learn from every execution to improve future decisions.

This progression from deterministic to adaptive behavior addresses the fundamental mismatch between static automation and dynamic distributed systems. As pipelines accumulate execution history, they develop institutional memory about flakiness patterns, dependency risks, regional stability, and optimal test strategies. Over time, the pipeline becomes progressively more effective at distinguishing signal from noise, avoiding known failure modes, and focusing validation effort where it matters most.

### 3.2 Five-layer architecture

**Figure 2:**
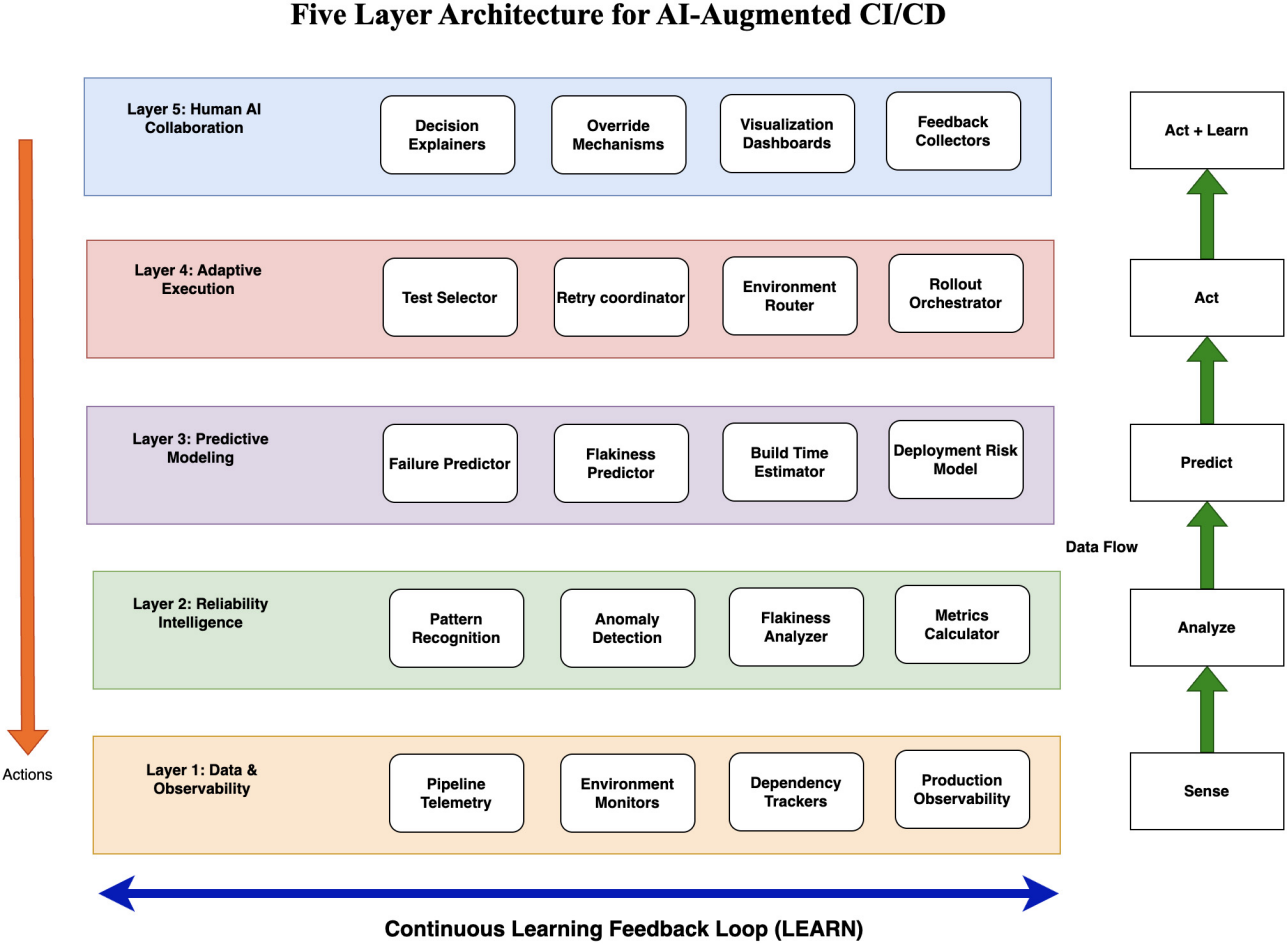
Five-Layer Architecture for AI-Augmented CI/CD. The architecture operationalizes the SAPAL loop. Layer 1 implements Sense, Layer 2 implements Analyze, Layer 3 implements Predict, Layer 4 implements Act, and Layer 5 implements Act and Learn through human collaboration. Data flows upward, actions flow downward, and feedback enables continuous learning.

The SAPAL loop provides a conceptual model for intelligent pipelines. This section presents a five-layer architectural framework that operationalizes the loop through concrete components. Each layer has a specific responsibility, and together they form a complete system for AI-augmented reliability. Figure 2 illustrates the layered architecture.

#### 3.2.1 Layer 1: Data and observability

The data and observability layer is the foundation of the architecture. It collects, stores, and provides access to all signals needed for intelligent decision making. This layer implements the sensing capability of the SAPAL loop.

Pipeline telemetry collectors capture data from every stage of the CI/CD workflow including code commits, build executions, test runs, artifact generation, deployment events, and production releases. Environment monitors track infrastructure conditions including runner availability, CPU and memory usage, network performance, storage I/O, and container health. Dependency trackers maintain an up-to-date view of all system dependencies including direct and transitive library versions, service API versions, and database schema versions.

Test result aggregators consolidate test outcomes from multiple sources and normalize data across different testing frameworks. Production observability integrations connect the pipeline to production monitoring systems, error tracking platforms, and incident management tools (Beyer et al., 2016). Historical data stores maintain long-term records of pipeline executions, test results, deployment outcomes, and incidents.

The data layer must be comprehensive without being overwhelming, real-time capable for adaptive pipelines, schema-flexible to accommodate evolution, and privacy-aware while collecting operational data.

#### 3.2.2 Layer 2: Reliability intelligence

The reliability intelligence layer processes data from Layer 1 to extract patterns, detect anomalies, and compute reliability metrics. This layer implements the analysis capability of the SAPAL loop.

Pattern recognition engines identify recurring failure modes, common error signatures, and temporal correlations using clustering algorithms, sequence mining, and association rule learning (Han et al., 2011). Anomaly detectors flag deviations from normal behavior using statistical methods, time series analysis, and unsupervised learning (Chandola et al., 2009). Flakiness analyzers characterize tests that fail intermittently and determine whether failures are timing dependent, environment dependent, or order dependent (Lam et al., 2019).

Dependency impact analyzers trace how changes propagate through the system and compute blast radius estimates (Cox, 2019). Root cause correlators link failures to their likely causes using causal inference techniques and domain-specific rules. Reliability metric calculators compute the metrics defined in Section 3.3.

The intelligence layer must be interpretable so developers can understand and act upon insights, actionable to directly inform predictions and actions, incremental to update continuously as new data arrives, and context-aware to consider the specific characteristics of each project and team.

#### 3.2.3 Layer 3: Predictive modeling

The predictive modeling layer uses analyzed data to forecast future outcomes. This layer implements the prediction capability of the SAPAL loop.

Failure prediction models forecast which tests are likely to fail for a given code change using features including changed files, affected modules, test execution history, and developer patterns (Bertolino et al., 2020). Common techniques include random forests, gradient boosting, and neural networks. Flakiness prediction models estimate the probability that a test will exhibit flaky behavior under current conditions (Pinto et al., 2020). Build time prediction models estimate how long a build will take (Hassan and Wang, 2017).

Deployment risk models assess the likelihood of rollback, error rate increase, or performance degradation by analyzing code complexity, change frequency, production history, and regional stability (Vassallo et al., 2019). Resource demand models predict CPU, memory, storage, and network requirements. Regional behavior models forecast how deployments will perform in different regions (Zhang et al., 2018).

Models are trained using historical data and evaluated using standard metrics including precision, recall, F1 score, and area under the ROC curve (Hastie et al., 2009). Models are retrained periodically to adapt to changing system behavior (Spieker et al., 2017). Predictions include confidence scores that reflect model certainty, with low-confidence predictions triggering conservative actions or human review.

The prediction layer must be accurate but robust, fast enough for real-time pipeline execution, explainable with contributing factors visible, and diverse with multiple models addressing different prediction tasks.

#### 3.2.4 Layer 4: Adaptive execution

The adaptive execution layer uses predictions to modify pipeline behavior at runtime. This layer implements the action capability of the SAPAL loop.

Test selectors choose which tests to run based on failure predictions and code change analysis, balancing coverage and speed (Yaraghi et al., 2023). Retry coordinators decide whether to retry failed tests based on flakiness predictions and failure signatures, implementing intelligent retry strategies including exponential backoff, environment switching, and retry budgets (Bell et al., 2018). Environment routers assign tests to execution environments that maximize reliability by considering environment stability, test requirements, and resource availability (Hilton et al., 2017).

Rollout orchestrators implement risk-based deployment strategies, determining canary sizes, rollout speeds, monitoring thresholds, and rollback triggers based on deployment risk predictions (Vassallo et al., 2019). Quality gate adjusters apply dynamic quality gates that balance safety and speed. Alert generators produce notifications only when failures are unexpected or high-confidence, filtering noise by suppressing alerts for known flaky tests.

The execution layer must be transparent with every decision logged, reversible so adaptive decisions can be overridden manually, safe so adaptive actions never compromise correctness, and performant so adaptation occurs in real time.

#### 3.2.5 Layer 5: Human-AI collaboration

The human-AI collaboration layer enables developers and operators to interact with the intelligent pipeline. This layer ensures that AI augments human intelligence rather than replacing it. It implements both the action and learning capabilities of the SAPAL loop.

Decision explainers provide human-readable explanations for pipeline actions, answering questions such as why a test was selected or why a region was flagged as risky. Override mechanisms allow humans to correct pipeline decisions when they have information the pipeline lacks. Overrides are logged and analyzed to improve future decisions.

Feedback collectors gather developer and operator input on pipeline behavior and integrate feedback into the learning process. Visualization dashboards present pipeline intelligence in accessible formats showing reliability metrics, prediction accuracy, flakiness trends, and regional stability. Configuration interfaces enable teams to customize pipeline behavior by setting risk tolerances, test selection policies, and rollout strategies. Incident review tools support post-mortem analysis of failures.

The collaboration layer must prioritize trust through transparency and responsiveness to feedback, respect human expertise rather than overriding it, be learnable so developers can quickly understand how to interact with the intelligent pipeline, and support continuous improvement through bidirectional human-AI collaboration.

#### 3.2.6 Layer interactions and data flow

The five layers form an integrated system. Data flows upward from Layer 1 through Layer 3, generating predictions. Predictions flow downward to Layer 4, triggering actions. Actions generate outcomes that flow back to Layer 1 as new data, closing the loop. Layer 5 spans all layers, providing human oversight and feedback at every stage.

The architecture supports incremental adoption, allowing organizations to implement layers gradually. It is technology-agnostic, describing responsibilities and interactions without prescribing specific tools. The architecture supports federation, enabling large organizations to deploy per team while sharing learned knowledge across deployments.

### 3.3 Reliability metrics for intelligent pipelines

Traditional CI/CD metrics focus on speed and throughput including build time, deployment frequency, lead time for changes, and mean time to recovery (Forsgren et al., 2018). These metrics are valuable but do not capture whether a pipeline is intelligent, adaptive, or improving over time. AI-augmented pipelines require new metrics that measure decision quality, learning effectiveness, and reliability intelligence.

This section proposes three core metrics for evaluating AI-augmented CI/CD systems. We provide theoretical grounding for each metric formulation and discuss how organizations should calibrate them for their specific contexts. All metrics are computed over a configurable time window such as 7 days, 30 days, or per release cycle. A rolling window captures recent trends while smoothing transient fluctuations.

#### 3.3.1 Pipeline health index

The Pipeline Health Index (PHI) measures overall system reliability by combining multiple reliability signals into a single composite score, as defined in Equation 1. PHI ranges from 0 to 1, where higher values indicate stronger pipeline reliability.


$$\begin{array}{l} \text{PHI}=w_{1}\cdot \text{SuccessRate}+w_{2}\cdot \left(1-\text{FlakinessRate}\right)+w_{3}\cdot \\ \text{ PredictionAccuracy}+w_{4}\cdot \left(1-\text{RollbackRate}\right) \end{array}$$ (1)

where *w*_*i*_ ≥ 0 and $\overset{4}{\underset{i=1}{\sum }}w_{i}=1$. All component metrics are normalized to the range [0, 1], ensuring bounded aggregation and interpretability. Each component is computed as:


$$\begin{array}{l} \text{SuccessRate}=\frac{\text{Builds Passing All Validation Stages}}{\text{Total Builds}}\\ \text{FlakinessRate}=\frac{\text{Flaky Test Failures}}{\text{Total Test Failures}}\\ \text{PredictionAccuracy}=F_{1}\left(\text{predictions},\text{actual outcomes}\right)\\ \text{RollbackRate}=\frac{\text{Rolled Back Deployments}}{\text{Total Deployments}} \end{array}$$

A build is considered successful if it completes all configured validation stages (e.g., automated tests, static analysis, security checks) without failure. Flaky test failures are identified using Test Stability Score (TSS) analysis. Prediction accuracy quantifies the quality of failure forecasting models. Rollback events capture deployments reversed due to errors, performance regressions, or failed health checks.

For example, given SuccessRate = 0.85, FlakinessRate = 0.15, PredictionAccuracy = 0.80, RollbackRate = 0.05, and default weights (*w*_1_, *w*_2_, *w*_3_, *w*_4_) = (0.3, 0.3, 0.2, 0.2):


$$\begin{array}{l} \text{PHI}=0.3\left(0.85\right)+0.3\left(1-0.15\right)+0.2\left(0.80\right)+0.2\left(1-0.05\right)\\ \;=0.255+0.255+0.160+0.190\\ \;=0.860 \end{array}$$

The PHI formula uses a weighted linear combination, a well-established approach for composite metrics in software engineering (Fenton and Bieman, 2014). We propose default weights of 0.3, 0.3, 0.2, 0.2 based on the following reasoning. SuccessRate and FlakinessRate receive higher weights because they directly impact developer productivity and are observable in every pipeline execution. PredictionAccuracy and RollbackRate receive lower weights because they depend on model maturity and deployment frequency, which vary across organizations.

Organizations should calibrate weights based on their priorities. Teams with high deployment frequency may increase *w*_4_ to emphasize rollback prevention. Teams with severe flakiness problems may increase *w*_2_ to prioritize stability. We recommend starting with default weights and adjusting based on quarterly reviews of which PHI components correlate most strongly with developer satisfaction and incident rates.

PHI serves as the top-level reliability indicator within the SAPAL loop. When PHI degrades, the system identifies which component contributed to the decline and adjusts its strategies accordingly, triggering targeted diagnostics, stricter test selection, or more conservative rollout policies.

#### 3.3.2 Test stability score

The Test Stability Score (TSS) quantifies how consistently a test produces the same outcome given identical code and comparable execution conditions, as defined in Equation 2. TSS ranges from 0 to 1, where 1 indicates perfect determinism and 0 indicates extreme flakiness. It provides a direct measure of behavioral stability and helps identify tests that introduce non-deterministic noise into the pipeline.


$$\begin{array}{l} \text{TSS}\left(t\right)=\frac{\text{ConsistentRuns}\left(t\right)}{\text{TotalRuns}\left(t\right)} \end{array}$$ (2)

where ConsistentRuns(*t*) counts executions in which test *t* produced identical outcomes across repeated executions on unchanged code, and TotalRuns(*t*) is the total number of executions during the measurement window on unchanged code. TSS therefore isolates non-deterministic behavior from consistent defect detection.

For example, consider a test executed 100 times on identical code that passes 95 times and fails 5 times without any code changes:


$$\text{TSS}\left(t\right)=\frac{95}{100}=0.95$$

TSS measures behavioral consistency, analogous to reliability estimation where component reliability is expressed as success probability over repeated trials (Fenton and Bieman, 2014). TSS is typically tracked over a rolling window (e.g., 50–200 executions) to reflect recent behavior. A threshold of 0.85 for identifying problematic tests is consistent with industry observations that tests failing more than 15 percent of the time due to non-determinism are considered unacceptably flaky (Lam et al., 2019).

Tests with TSS < 0.85 are candidates for isolation or repair (Lam et al., 2019). TSS informs retry policies in the adaptive execution layer. High-TSS tests (TSS>0.98) fail without retry to provide rapid feedback on likely deterministic regressions. Low-TSS tests receive bounded intelligent retry under improved conditions (e.g., increased timeout or isolated execution). This approach reduces false failures while preserving rapid detection of genuine defects.

#### 3.3.3 Failure prediction confidence

Failure Prediction Confidence measures the accuracy and reliability of predictive models used in the analysis and prediction layers. FPC combines classification quality and probability reliability into a single measure between 0 and 1, as defined in Equation 3.


$$\begin{array}{l} \text{FPC}=\alpha \cdot F_{1}+\beta \cdot \text{Calibration} \end{array}$$ (3)

*F*_1_ is the harmonic mean of precision and recall, capturing correctness of binary failure predictions. Calibration is the degree to which predicted probabilities match actual failure frequencies, measured using Expected Calibration Error (Niculescu-Mizil and Caruana, 2005). α and β are weights that balance discrimination and calibration.

FPC combines two complementary aspects of prediction quality. F1 score measures discrimination, the ability to correctly classify failures versus successes. Calibration measures probability accuracy, ensuring that when a model predicts 80 percent failure probability, approximately 80 percent of such cases actually fail. Both properties are essential for reliable decision making in adaptive systems (Niculescu-Mizil and Caruana, 2005). We propose default weights of α = 0.7 and β = 0.3 because discrimination has more direct impact on test selection decisions, while calibration becomes critical when setting confidence thresholds for automation.

Organizations with conservative cultures may increase β to emphasize probability accuracy, reducing the risk of over-optimistic predictions. Organizations prioritizing throughput may increase α to focus on classification accuracy.

High FPC above 0.80 indicates that predictions are both accurate and well-calibrated. When FPC is high, the system may skip tests that are predicted to pass with high probability. When FPC deteriorates below 0.75, the system becomes conservative and expands testing until model accuracy improves.

FPC governs how much trust the pipeline places in predictive models. High FPC enables optimization such as test skipping and targeted prioritization, while low FPC triggers fallback strategies such as broader test coverage or disabling risk-based actions.

### 3.4 Decision logic and safety guarantees

With the SAPAL loop, architecture, and metrics defined, we now formalize the decision logic that guides adaptive pipeline behavior. Algorithm 1 operationalizes the Act stage of the SAPAL loop, demonstrating how predictions guide adaptive execution while maintaining strict safety guarantees.

Algorithm 1 SAPAL act stage: safety-first decision logic.

Require: Test *t*, CodeChange *c*, PredictionModel *M*
Ensure: Test execution decision and result
1: // Sense and Analyze: Gather intelligence signals
2: *tss*← ComputeTestStabilityScore(*t*)
3: *drs*← ComputeDependencyRiskScore(*t*, *c*)
4: *fpc*← ModelConfidence(*M*)
5: // Predict: Forecast test outcome
6: (*prediction, confidence*)←*M*.Predict(*t*, *c*)
7: // Act: Safety-first execution decision
8: if CanSafelySkip(*drs*, *tss*, *prediction*, *confidence*, *fpc*) then
9:   skip test *t*
10:    return *SKIPPED*
11: end if
12: // Execute: Run test with intelligent failure handling
13: *result*← RunTest(*t*)
14: if *result* = *FAIL* then
15:   if *tss* < 0.85 then
16:    Apply intelligent retry with improved conditions
17:    if retry succeeds then
18:      return *RECOVERED*
19:    else
20:      return *FAIL*
21:     end if
22:   else
23:      return *FAIL*
24:    end if
25: else
26:     return *PASS*
27: end if

#### 3.4.1 Safety-first design principles.

SAPAL enforces safety constraints that dominate optimization objectives. A critical concern is whether an adaptive pipeline might learn to skip tests that fail frequently. The framework prevents this through defense in depth.

First, Test Stability Score (TSS) measures determinism on identical code and comparable execution conditions, not overall failure frequency. Stability alone is never sufficient for skipping. The CanSafelySkip() function (Algorithm 1, line 8) permits skipping only when dependency risk is low, TSS is very high (e.g., >0.98), the predicted failure probability for the current change is very low (e.g., *p*_fail_ < 0.01), per-decision confidence is very high (e.g., >0.99), and overall model quality is strong (e.g., *FPC*>0.85). All conditions must be satisfied simultaneously.

Second, SAPAL never skips tests with recent failures in the last *N* comparable executions (e.g., *N* = 10), regardless of prediction confidence. Third, the system differentiates flaky tests (low TSS) from stable tests. Flaky failures trigger bounded intelligent retry under improved execution conditions, whereas stable tests fail without retries, surfacing likely deterministic regressions quickly.

Additional safety rails prevent optimization from compromising correctness. Organizations may enforce skip-rate bounds (e.g., 10–20% of the test suite), mandate periodic full test runs (nightly or pre-release) to validate adaptive behavior, and designate critical tests that override adaptive decisions. Human override capability with audit logging provides a final safeguard. When prediction confidence or model quality is insufficient, the system defaults to conservative execution with broader test coverage. This defense-in-depth design ensures that uncertainty resolves toward safety rather than speed.

The algorithm implements a conservative approach to test optimization. The CanSafelySkip function enforces three independent safety conditions before allowing test bypass. Dependency risk must be low. Test stability must be very high with TSS greater than 0.98. Prediction confidence must be very high with confidence greater than 0.99 and sufficient model quality with FPC greater than 0.85. This ensures that only demonstrably stable, low-risk tests with very confident predictions are skipped.

For tests that execute and fail, the algorithm distinguishes between flaky patterns with low TSS and high-confidence bugs. Flaky tests receive intelligent retry with improved conditions such as increased timeout, isolated environment, or fresh state. High-confidence bugs fail fast to provide immediate developer feedback. Real bugs persist through retry attempts because they represent deterministic code defects rather than environmental instability, ensuring comprehensive bug detection while eliminating flaky noise.

This safety-first design reflects SAPAL's core principle. Aggressive noise reduction must never compromise correctness. The three-layer validation system provides defense in depth, requiring multiple independent confirmations before optimization.

## 4 Framework application

This section demonstrates how the SAPAL framework applies to real CI/CD reliability challenges through three application scenarios. Each scenario is grounded in patterns and parameters documented in published research. We walk through the SAPAL loop stages to show how the framework operates end to end. We project expected outcomes using empirical findings from the literature. These projections represent hypothetical improvements based on component studies rather than measured results from integrated framework deployment. The scenarios illustrate framework applicability while clearly distinguishing literature-grounded demonstrations from empirical validation.

### 4.1 Flaky test management

Flaky tests represent a persistent challenge in CI/CD pipelines. These tests pass or fail intermittently without any code changes, typically due to timing issues, resource contention, or environment variability (Luo et al., 2014). Research shows that flaky tests affect 11–27 percent of tests in large industrial projects (Lam et al., 2019) and account for 5 to 16 percent of build failures (Luo et al., 2014). Thorve et al. (Thorve et al., 2018) found flaky test rates as high as 45 percent in certain Android projects.

#### 4.1.1 Scenario context

Consider a representative scenario based on industrial scale reported in CI/CD studies (Hilton et al., 2017; Forsgren et al., 2018). A development team maintains a microservices platform with approximately 10,000 integration tests. Based on the flaky test rates reported by Lam et al. (Lam et al., 2019), approximately 500 tests exhibit flaky behavior. Using the build failure rates from Luo et al. (Luo et al., 2014), approximately eight percent of builds fail due to these flaky tests, blocking pull requests and consuming developer time for investigation.

#### 4.1.2 SAPAL loop application

Sense. The Data and Observability Layer collects signals from each test execution. Based on observability practices described by Beyer et al. (2016), the layer captures test execution history including pass or fail outcomes for each test across a rolling window. Lam et al. (2019) recommend windows of 100–200 executions to capture flaky patterns. The layer also captures execution timing including mean and standard deviation of execution time per test, enabling detection of timing-sensitive tests as described by Dutta et al. (2020). Infrastructure metrics including CPU utilization, memory pressure, and network latency on test runners follow the infrastructure monitoring approach of Hilton et al. (2017). Failure signatures including stack traces and error messages are categorized using the failure taxonomy from Luo et al. (2014).

Analyze. The Reliability Intelligence Layer processes collected data to identify flaky tests and characterize their failure modes. For each test, the layer computes Test Stability Score using the formula from Section 3.3.2. Following the threshold recommendations from Lam et al. (2019), tests with TSS below 0.85 are flagged as potentially flaky. In this scenario, the layer identifies approximately 500 tests meeting this criterion.

The layer categorizes flaky tests using the taxonomy from Luo et al. (2014). Async wait failures account for 28 percent of flaky tests, where tests fail due to insufficient wait times for asynchronous operations. Concurrency failures account for 21 percent, where tests fail due to race conditions or thread synchronization issues. Test order dependency accounts for 12 percent, where tests fail when executed in certain orders relative to other tests. Resource leak accounts for 11 percent, where tests fail due to accumulated resource exhaustion across test runs. Other causes including network issues, time dependencies, floating point precision, and unordered collections account for 28 percent. These percentages are derived from the empirical analysis of 201 flaky tests by Luo et al. (2014).

Predict. The Predictive Modeling Layer forecasts which tests are likely to exhibit flaky behavior in the current build. Following the hybrid approach of Parry et al. (2023), the model combines historical flakiness patterns with current execution context. Input features are based on features identified as predictive by Bell et al. (2018) and Pinto et al. (2020). These include historical TSS for each test, recent failure rate in the last 10 executions, test execution time relative to historical mean, current infrastructure load metrics, and time since last environment refresh.

For each test, the model produces a flakiness probability between 0 and 1. Parry et al. (2023) report that ML models achieve precision of 73–91 percent for flaky test prediction depending on the dataset and approach. In this scenario, the model flags 15 tests with flakiness probability greater than 0.70 for the current build based on historical patterns and current infrastructure conditions.

Act. The Adaptive Execution Layer implements response strategies based on predictions. Following the intelligent retry approaches evaluated by Parry et al. (2023) and Bell et al. (2018), the layer takes three actions.

Action 1 quarantines high-risk tests. Tests with flakiness probability greater than 0.70 are moved to a separate non-blocking suite. Their results are reported but do not block the build, following the quarantine strategy described by Lam et al. (2019).

Action 2 applies intelligent retry policy. For quarantined tests, the pipeline applies retry logic based on failure mode. Async wait failures receive retry with increased timeout of 1.5 × to 2 × , as recommended by Luo et al. (2014). Concurrency failures receive retry up to 3 times, consistent with the rerun strategies evaluated by Parry et al. (2023). Resource leak failures receive retry in isolated environment with fresh state.

Action 3 performs environment routing. Tests classified as environment-sensitive are routed to runners with lower utilization, following the infrastructure-aware approach of Hilton et al. (Hilton et al., 2017).

Learn. The Human-AI Collaboration Layer presents results and collects feedback to improve future predictions. After build completion, 15 tests were flagged as potentially flaky. Of these, 12 tests passed after retry, confirming flaky behavior. Three tests failed consistently and were flagged for developer review.

Developers review flagged tests and provide corrections. Confirmed classifications reinforce model weights. Corrections become training examples for model improvement. Model parameters are updated based on outcomes, following the reinforcement learning approach of Spieker et al. (2017). Over time, prediction accuracy improves as the model learns project-specific flakiness patterns.

#### 4.1.3 Projected outcome

Based on empirical results from related studies, Bell et al. (2018) report that DeFlaker identifies flaky tests with 96 percent precision and 61 percent recall. Parry et al. (2023) report that CANNIER achieves similar accuracy with 10 × faster detection. For failure resolution, Parry et al. (2023) found that intelligent retry strategies resolve 70–90 percent of flaky test failures when combined with accurate detection. Lam et al. (Lam et al., 2019) report that flaky test investigation consumes significant developer effort. Automated detection and resolution can substantially reduce this overhead.

For this scenario, applying detection precision of 80 percent and retry success rate of 75 percent, the projected reduction in false build failures is 4.8 percent. This would reduce the flaky-induced build failure rate from 8 percent to approximately 3.2 percent, representing a 60 percent improvement in build stability attributable to flaky test management. This projection assumes baseline conditions from Luo et al. (2014) (8% flaky-induced failure rate), detection precision of approximately 80% (Bell et al., 2018; Parry et al., 2023), and intelligent retry success rate of 75% (Parry et al., 2023). Actual outcomes depend on data quality, model calibration accuracy, and infrastructure stability. See Section 4.4 for validation considerations.

### 4.2 Selective test execution

Large codebases accumulate extensive test suites that become impractical to run for every change. Hilton et al. (2017) found that CI build times in open source projects range from minutes to hours, with test execution accounting for the majority of build time. Zhao et al. (2024) note that running complete test suites for every commit creates feedback delays that reduce developer productivity.

#### 4.2.1 Scenario context

Consider a monorepo containing 40 services with a combined test suite of 15,000 tests, consistent with the scale reported in industrial CI/CD studies (Forsgren et al., 2018; Hilton et al., 2017). Running all tests takes over an hour. Most commits affect only 1–3 services.

#### 4.2.2 SAPAL loop application

Sense. The pipeline collects code change metadata including files modified, modules affected, and historical test-to-code mappings following the dependency analysis approach of Zhang et al. (2021).

Analyze. The Reliability Intelligence Layer computes historical failure correlations between code changes and test outcomes, identifying which tests are most likely to detect faults in specific modules.

Predict. Using ML-based test prioritization techniques surveyed by Pan et al. (2021), the model ranks tests by failure probability for the current change. Zhao et al. (2024) report that pretrained models achieve optimal test ordering on 80 percent of subjects.

Act. The pipeline executes high-priority tests immediately, the top 20 percent by predicted failure probability, and defers low-priority tests to post-merge execution. Spieker et al. (2017) report that RETECS reduces test execution time while maintaining fault detection effectiveness.

Learn. When deferred tests detect faults, the model is updated to increase their priority for similar future changes.

#### 4.2.3 Projected outcome

Based on empirical evaluations of ML-based test selection, Spieker et al. (2017) report significant reductions in test execution time while maintaining fault detection rates. Zhao et al. (2024) demonstrate that cross-project pretraining improves prioritization effectiveness from 50–80 percent optimal orderings. Pan et al. (2021) survey reports reductions in feedback time of 50–80 percent across multiple studies.

For this scenario, applying conservative estimates from these studies, selective execution could reduce feedback time by 50–80 percent for typical changes while maintaining high fault detection rates. These estimates assume mature test-to-code mappings, stable change patterns, and sufficient execution history for training. Organizations with limited historical data or high code churn may experience more modest initial improvements. See Section 4.4 for validation considerations.

### 4.3 Regional deployment strategies

Global services deploy across multiple regions with different infrastructure characteristics, traffic patterns, and operational conditions. (Vassallo et al., 2019) found that deployment failures vary significantly across environments, with certain configurations experiencing higher failure rates.

#### 4.3.1 Scenario context

Consider a service deployed to 6 regions. Historical data shows deployment success rates varying across regions, with some regions experiencing elevated rollback rates due to infrastructure instability. This pattern is consistent with the environmental variability documented in CI/CD studies (Vassallo et al., 2019; Hilton et al., 2017).

#### 4.3.2 SAPAL loop application

Sense. The pipeline collects regional deployment history including canary success rates, error rates, latency metrics, and rollback frequency, following observability practices from Beyer et al. (2016).

Analyze. The Reliability Intelligence Layer computes regional stability scores and identifies patterns such as time-of-day effects, traffic correlation, and infrastructure events that predict deployment risk.

Predict. The model estimates deployment success probability for each region based on current conditions. Vassallo et al. (2019) demonstrate that build and deployment outcomes can be predicted with reasonable accuracy using historical features.

Act. The rollout orchestrator applies region-specific strategies. High-confidence regions with predicted success greater than 95 percent receive standard canary size of 5 percent with normal observation period. Medium-confidence regions with 85 to 95 percent predicted success receive smaller canary of 2 percent with extended observation. Low-confidence regions with predicted success below 85 percent receive minimal canary of 1 percent with strict thresholds and manual approval gate.

Learn. Deployment outcomes update regional stability models and refine prediction accuracy over time.

#### 4.3.3 Projected outcome

Vassallo et al. (2019) found that deployment failures often correlate with identifiable risk factors. By applying differentiated rollout strategies based on predicted risk, organizations can reduce overall rollback rates while maintaining deployment velocity in stable regions. The specific improvement depends on baseline failure rates and the accuracy of risk prediction in each organizational context. Actual benefit magnitude depends on baseline regional variance, infrastructure heterogeneity, and prediction model accuracy. Organizations with consistently high regional stability may see minimal incremental improvement. See Section 4.4 for validation considerations.

### 4.4 Illustrative walkthrough

To illustrate SAPAL's operational logic, we present a compact example of a single CI/CD build. This example is analytical and intended to demonstrate internal consistency rather than empirical validation.

A developer modifies payment_processor.py and payment_validator.py. Historical metrics show:


$$\text{TSS}=\{\begin{array}{l} 0.95\text{ payment\_processor\_test}\\ 0.72\text{ database\_integration\_test}\\ 0.98\text{ payment\_validator\_test} \end{array}$$

Current infrastructure load is moderate (CPU 65%, memory 70%). The predictive model estimates failure probabilities of 0.15, 0.45, and 0.05 respectively, with confidence above 0.88 and *FPC* = 0.87.

Following Algorithm 1:

- payment_processor_test is executed due to moderate predicted risk.
- database_integration_test is executed with bounded retry enabled because TSS < 0.85.
- payment_validator_test is evaluated for skipping. Although TSS = 0.98, per-decision confidence does not exceed the required safety threshold (>0.99), so the test is executed.

During execution, database_integration_test fails initially but passes on retry under improved conditions, indicating flakiness. The system updates its stability estimate (0.72 → 0.71) and records the retry outcome for future model refinement.

This walkthrough demonstrates how SAPAL integrates prediction, stability metrics, and safety constraints to mitigate non-deterministic failures while preserving detection of deterministic defects.

### 4.5 Validation considerations

The application scenarios in this section demonstrate framework applicability using literature-grounded scenarios and published empirical metrics. While projected outcomes are based on peer-reviewed findings, they represent expected improvements rather than measured results from framework deployment.

Rigorous empirical validation would require prototype implementation building functional components for the five architectural layers, baseline measurement establishing current CI/CD reliability metrics before framework deployment, controlled comparison measuring the same metrics after framework deployment to quantify actual improvements, longitudinal study evaluating learning effectiveness by tracking prediction accuracy and adaptation quality over weeks to months, multiple organizations assessing generalizability across different organizational contexts, team sizes, and technology stacks, and A/B testing comparing SAPAL-augmented pipelines against baseline pipelines in production environments.

The literature-grounded approach used in these scenarios provides reasonable expectations for framework benefits based on improvements demonstrated in component studies. Actual outcomes will depend on implementation quality, organizational context, data availability, and integration with existing CI/CD infrastructure. Organizations with mature observability practices and substantial historical data are more likely to achieve results toward the upper end of projected ranges, while organizations with limited data may experience more modest improvements initially, with performance improving as the system accumulates learning data.

We identify empirical validation through industrial deployment as the most important direction for future work. The framework provides a conceptual foundation and architectural blueprint. Translating this into production systems and measuring real-world impact represents the critical next step for this research.

## 5 Discussion

This section discusses the practical implications of the AI-augmented reliability framework for research and industry. We examine how organizations can adopt the framework incrementally, address implementation challenges, and align the approach with existing CI/CD practices. We also acknowledge limitations of the current work and identify areas requiring further investigation.

### 5.1 Implications for practice

The SAPAL framework introduces a structured approach for integrating AI capabilities into CI/CD pipelines. Unlike point solutions that address isolated problems, the framework provides a comprehensive model that spans data collection, pattern analysis, predictive forecasting, adaptive execution, and continuous learning. This holistic approach enables organizations to address multiple reliability challenges within a unified system rather than deploying disconnected tools.

The five-layer architecture supports incremental adoption. Organizations can begin by enhancing observability capabilities in Layer 1, establishing the data foundation needed for intelligent decision making. They can then add reliability intelligence in Layer 2 to identify patterns and compute metrics. As confidence grows, they can introduce predictive models in Layer 3 and enable adaptive execution in Layer 4. Layer 5 ensures human oversight throughout this evolution. This gradual implementation path reduces risk and allows teams to validate benefits at each stage before proceeding.

The framework shifts pipeline evaluation from speed-focused metrics to reliability-focused metrics. Traditional metrics such as build time and deployment frequency remain important for measuring throughput. However, the three proposed metrics, Pipeline Health Index, Test Stability Score, and Failure Prediction Confidence, provide complementary measures of decision quality and learning effectiveness. Organizations can use these metrics to track whether their pipelines are becoming more intelligent over time, making more accurate predictions, and improving reliability outcomes.

The explicit inclusion of human-AI collaboration in Layer 5 acknowledges that pipeline intelligence should augment rather than replace human judgment. Developers retain the ability to override decisions, provide feedback, and guide system behavior. This collaborative approach builds trust and ensures that automation respects human expertise. Research on human-AI interaction shows that systems providing transparency and control achieve higher adoption rates than fully automated black-box systems (Amershi et al., 2019).

### 5.2 Adoption considerations

Organizations considering AI-augmented CI/CD should evaluate several factors before implementation. Data availability represents a fundamental prerequisite. The framework requires historical pipeline execution data, test results, deployment outcomes, and production metrics. Organizations with mature CI/CD practices typically have this data available through existing observability infrastructure. Organizations with limited historical data should focus initially on establishing comprehensive data collection before attempting to build predictive models.

Technical expertise requirements span multiple domains. Implementing the framework requires knowledge of software engineering, machine learning, and DevOps practices. Organizations should assess whether they have personnel with these skills or plan to develop them through training and hiring. Alternatively, organizations can partner with vendors offering AI-augmented CI/CD platforms that implement framework principles.

Cultural readiness matters as much as technical capability. AI-augmented pipelines represent a shift from deterministic automation to probabilistic decision making. Teams must be comfortable with predictions that include confidence scores rather than absolute certainty. This requires establishing feedback mechanisms, blameless post-mortems that treat pipeline errors as learning opportunities, and clear escalation paths when automated decisions require human review (Forsgren et al., 2018).

Integration with existing infrastructure determines implementation complexity. The framework must connect with current CI/CD platforms, testing frameworks, deployment tools, and monitoring systems. Organizations using modern, API-driven infrastructure will find integration easier than those with legacy systems. The layered architecture supports gradual integration, allowing organizations to connect one layer at a time rather than requiring wholesale replacement of existing tools.

Cost-benefit analysis should consider both direct costs and operational savings. Direct costs include compute resources for model training, storage for historical data, and engineering effort for implementation and maintenance. Benefits include reduced time spent investigating false failures, faster feedback from selective test execution, fewer production incidents from improved deployment strategies, and increased developer productivity from more reliable pipelines. Organizations should project these costs and benefits based on their current pain points and development scale.

### 5.3 Limitations and challenges

The framework presented in this paper is conceptual rather than empirical. We have not implemented a complete system or measured its performance in production environments. The application scenarios demonstrate feasibility using literature-grounded parameters, but they do not constitute validation. Empirical studies are required to confirm whether the framework delivers projected benefits across diverse organizational contexts.

#### 5.3.1 Conceptual and empirical limitations

As a theory-informed framework, SAPAL formalizes architectural principles and predictive control mechanisms without presenting integrated experimental validation. Projected improvements are extrapolated from component studies rather than measured end-to-end deployments, and should be interpreted as hypotheses rather than observed outcomes. Future work must evaluate SAPAL through controlled comparisons, longitudinal industrial studies, and A/B testing in production CI/CD environments.

#### 5.3.2 Technical and operational challenges

Implementing adaptive CI/CD systems introduces several technical and organizational challenges.

##### 5.3.2.1 Cold start and sparse data

New services with limited execution history may produce poorly calibrated models, leading to either over-cautious behavior (reduced velocity) or under-cautious behavior (missed defects). Mitigation includes extended observation phases before enabling adaptive features, transfer learning from related projects (Zhao et al., 2024), and human oversight during early deployment phases.

##### 5.3.2.2 Concept drift

Software systems evolve through refactoring, architectural changes, and technology migrations (Gama et al., 2014). Historical patterns may become obsolete, degrading prediction quality. Mitigation includes scheduled retraining, monitoring of reliability metrics such as FPC and PHI, feature distribution analysis, and manual model recalibration following major architectural transitions.

#### 5.3.3 Failure modes and reliability risks

Any adaptive system that modifies validation intensity must explicitly account for the risk of under-validation. SAPAL faces several scenarios where adaptive behavior could compromise reliability despite built-in safeguards.

##### 5.3.3.1 Miscalibrated prediction models

If models predict test passage with high confidence but actual pass rates are substantially lower, excessive test skipping may allow undetected defects to reach production. Mitigation includes regular monitoring of model quality metrics, conservative fallback policies when prediction reliability degrades, and mandatory periodic full test runs that bypass adaptive decisions.

##### 5.3.3.2 Adversarial or gaming behavior

Developers may learn patterns that inadvertently or deliberately bypass validation, such as decomposing substantive changes into multiple small commits that individually appear low-risk but cumulatively introduce defects. Mitigation includes independent code review processes, detection of unusual commit fragmentation patterns, and periodic audits of adaptive behavior.

##### 5.3.3.3 Cascade failures in multi-region deployment

Models may learn that a region is historically stable and deploy aggressively, while infrastructure conditions change rapidly. Mitigation includes region-specific rollout controls, anomaly detection in deployment metrics, and circuit breakers that halt rollout when safety thresholds are exceeded.

##### 5.3.3.4 Model bias and uneven validation coverage

Bias can emerge when historical training data reflects imbalanced validation practices. For example, modules that historically received limited testing may be deprioritized by predictive models, reinforcing gaps in coverage. Organizations should monitor prediction distribution across modules and teams and conduct periodic validation distribution audits to prevent systematic under-validation.

#### 5.3.4 Organizational and regulatory constraints

##### 5.3.4.1 Increased system complexity.

AI-augmented pipelines introduce probabilistic decision logic that can be harder to debug than static pipelines. The framework addresses this through explainability and audit mechanisms (Layer 5), but organizations must invest in tooling and training to maintain transparency and trust.

##### 5.3.4.2 Regulatory compliance

Certain industries require deterministic and auditable quality controls. Organizations in regulated sectors must carefully evaluate which pipeline stages may employ adaptive behavior and which must remain deterministic. The layered architecture supports selective adoption to accommodate regulatory constraints.

##### 5.3.4.3 Resource overhead

Training and running predictive models introduce computational overhead. Organizations must ensure that prediction latency and resource consumption do not negate the time savings gained from adaptive execution.

##### 5.3.4.4 General principle

SAPAL is designed to fail safely. When uncertainty increases or model quality degrades, the system defaults to broader validation, stricter execution policies, and human review rather than optimizing for speed. This conservative bias ensures that adaptive behavior cannot override correctness under uncertain or evolving conditions.

### 5.4 Comparison with existing approaches

The SAPAL framework differs from existing research and industry tools in several fundamental ways. Previous work addresses specific problems such as test prioritization, failure prediction, or flaky test detection in isolation. The framework integrates these capabilities within a unified adaptive loop that spans the entire delivery workflow.

Existing ML applications in CI/CD typically operate as separate tools that provide recommendations but do not directly control pipeline execution. The framework embeds intelligence within the pipeline itself through Layer 4, enabling automated adaptive decisions while preserving human oversight through Layer 5.

Classical adaptive system models such as MAPE-K provide valuable reference architectures but require modification for CI/CD contexts. The SAPAL loop explicitly addresses short-lived executions, multi-stage causality, inter-run learning, and human collaboration. These adaptations make the framework more applicable to software delivery than generic autonomic computing models.

Industry tools such as Launchable, Harness, and DataDog demonstrate practical value of AI in specific pipeline stages. The framework complements these tools by providing a conceptual model that explains how individual capabilities should integrate into a coherent system. Organizations can use the framework to evaluate existing tools and identify gaps in their current approach.

### 5.5 Research opportunities

The framework opens several directions for future research. Empirical validation through implementation and evaluation in real world environments represents the most immediate priority. Researchers and practitioners should build systems based on the framework and measure their impact on reliability, efficiency, and developer experience. Controlled experiments comparing SAPAL-augmented pipelines against baseline pipelines using A/B testing methodologies would provide valuable evidence.

Explainable AI techniques adapted specifically for CI/CD contexts deserve focused attention. Developers and operators must understand pipeline decisions to trust them. Techniques such as LIME and SHAP require adaptation for CI/CD data structures and workflows (Ribeiro et al., 2016; Lundberg and Lee, 2017). Research should explore how to present explanations in forms that developers find actionable and comprehensible.

Benchmark datasets and evaluation frameworks would enable rigorous comparison of different approaches. Current flaky test datasets address only one aspect of CI/CD reliability. Comprehensive benchmarks covering test prioritization, deployment risk, and dependency management would accelerate research progress. These benchmarks should include realistic scale, diverse technology stacks, and ground truth labels for evaluation.

Cross-organizational learning through privacy-preserving federated learning could amplify the framework's impact (Yang et al., 2019). Smaller projects could benefit from patterns learned in larger organizations while preserving proprietary information. Research should explore which knowledge transfers effectively across organizational boundaries and which patterns remain organization-specific.

Integration with emerging technologies such as foundation models for code understanding and autonomous software agents presents opportunities for enhanced capabilities (Chen et al., 2021). Large language models trained on code could improve test generation, failure diagnosis, and fix suggestion. Research should investigate how these capabilities integrate with the SAPAL loop and whether they provide incremental value over existing ML techniques.

### 5.6 Why conceptual framework first

Conceptual frameworks serve as essential prerequisites for rigorous empirical evaluation. This progression mirrors how foundational models in software engineering achieved acceptance. The MAPE-K autonomic computing model (Kephart and Chess, 2003) was introduced conceptually before large-scale validation studies appeared years later. The DORA metrics for DevOps performance (Forsgren et al., 2018) began as a conceptual framework before becoming empirically validated standards.

Three factors make a conceptual framework the appropriate first contribution for AI-augmented CI/CD. First, existing empirical studies validate individual components in isolation, such as test prioritization (Spieker et al., 2017), flaky test detection (Bell et al., 2018), and failure prediction (Saidani et al., 2022). No prior work demonstrates end-to-end integration of these capabilities. A unifying framework is required before such integration can be systematically evaluated.

Second, reproducible empirical evaluation requires shared conceptual vocabulary and evaluation criteria. Without agreement on what constitutes an intelligent pipeline, which metrics measure intelligence, and how components should integrate, empirical studies remain incomparable. The SAPAL framework, five-layer architecture, and three reliability metrics provide this foundation.

Third, industrial adoption requires architectural blueprints before implementation. Organizations cannot build intelligent pipelines from disconnected research papers. The framework provides a reference architecture that guides implementation while supporting incremental adoption and technology-agnostic deployment.

Empirical validation remains the critical next step, as emphasized in Section 6.1. However, that validation builds upon rather than replaces the conceptual foundation established here.

## 6 Conclusion

Modern software delivery faces a fundamental challenge. CI/CD pipelines operate as fixed, deterministic scripts while the systems they support behave as dynamic, distributed ecosystems. This mismatch creates reliability problems including flaky tests, environment instability, dependency drift, pipeline noise, and regional deployment variations. Traditional approaches based on static rules and manual intervention cannot keep pace with increasing system complexity and delivery velocity.

This paper proposes a comprehensive framework for AI-augmented CI/CD reliability that transforms pipelines from deterministic automation into adaptive, intelligent systems. The framework consists of four integrated components that work together to enable continuous improvement in pipeline behavior.

The Sense-Analyze-Predict-Act-Learn loop provides the conceptual foundation. This loop extends classical adaptive system models such as MAPE-K with CI/CD-specific constructs including multi-region deployment awareness, dependency risk quantification, flakiness characterization, and developer feedback integration. The loop operates continuously across pipeline executions, sensing real-world conditions, analyzing patterns, predicting outcomes, adapting execution strategies, and learning from results. By making prediction an explicit first-class stage and incorporating domain-specific knowledge, SAPAL addresses the unique requirements of software delivery contexts that differ fundamentally from long-running autonomic systems.

The five-layer architectural framework operationalizes the SAPAL loop through concrete components and clear interfaces. Layer 1 handles data collection and observability. Layer 2 performs reliability intelligence and pattern recognition. Layer 3 implements predictive modeling and forecasting. Layer 4 executes adaptive decisions including test selection, intelligent retry, and risk-based rollout. Layer 5 enables human-AI collaboration through explanations, overrides, and feedback mechanisms. Each layer addresses specific technical challenges while maintaining modularity that supports incremental adoption.

Three core reliability metrics shift evaluation focus from raw speed to meaningful reliability and continuous improvement. Pipeline Health Index measures overall system reliability by combining success rate, flakiness rate, prediction accuracy, and rollback rate. Test Stability Score quantifies test consistency to identify flaky behavior and guide retry policies. Failure Prediction Confidence evaluates the accuracy and calibration of predictive models to determine appropriate trust levels for automated decisions. These metrics provide the instrumentation needed to assess whether pipelines are learning effectively and improving over time.

Three application scenarios demonstrate how the framework addresses real CI/CD reliability challenges. Flaky test management uses intelligent retry and quarantine strategies grounded in failure mode classification. Empirical studies suggest this approach can resolve 70 to 90 percent of flaky failures automatically. Selective test execution applies risk-based prioritization to reduce feedback time by 50 to 80 percent while maintaining fault detection effectiveness. Regional deployment implements stability-aware rollout orchestration that adapts strategies based on predicted regional risk. These scenarios illustrate framework applicability while acknowledging that rigorous empirical validation through industrial deployment remains essential future work.

### 6.1 Future work

As outlined in Section 5.5, the most critical direction for future research is empirical validation through implementation and evaluation in real-world environments. This requires several specific activities.

Prototype implementation across multiple organizations and technology stacks will reveal practical challenges and inform framework refinement. Organizations should build functional components for the five architectural layers and integrate them with existing CI/CD infrastructure. Implementation experiences will identify which components provide the most value, which prove most difficult to build, and how the framework should adapt to different organizational contexts.

Controlled experimentation using A/B testing methodologies will quantify framework benefits. Organizations should compare SAPAL-augmented pipelines against baseline pipelines on identical codebases and measure differences in reliability metrics, developer productivity, and incident rates. Baseline measurements taken before framework deployment will establish current performance levels. Longitudinal studies tracking metrics over months will assess whether learning mechanisms deliver sustained improvements.

Multi-organizational studies will evaluate generalizability. The framework should be tested across different team sizes, technology stacks, deployment frequencies, and organizational cultures. Comparing results across organizations will reveal which aspects of the framework transfer broadly and which require customization for specific contexts.

Beyond empirical validation, key research opportunities include explainable AI techniques adapted for CI/CD contexts to help developers understand and trust pipeline decisions, benchmark datasets covering test prioritization, deployment risk, and dependency management to enable systematic comparison of different approaches, privacy-preserving federated learning to enable cross-organizational knowledge sharing, and integration with foundation models for code understanding to enhance test generation and failure diagnosis capabilities.

### 6.2 Closing statement

The framework presented in this paper addresses critical gaps in current CI/CD practice and research. By integrating predictive analytics, adaptive decision-making, and continuous learning within a unified conceptual model grounded in adaptive systems theory but adapted for software delivery contexts, it provides a structured approach for building intelligent pipelines. The SAPAL loop, five-layer architecture, and reliability metrics establish both a theoretical foundation and practical blueprint for organizations seeking to improve CI/CD reliability at scale.

As organizations continue to embrace multi-region architectures, expand microservice ecosystems, and accelerate delivery velocity, the limitations of static pipelines become increasingly apparent. Larger systems generate more noise. More dependencies introduce more failure modes. More regions create more behavioral variations. In this environment, the ability to learn from experience, predict risks accurately, and adapt strategies intelligently becomes essential rather than optional.

The path forward requires collaboration between researchers and practitioners. Researchers should focus on empirical validation, algorithm development, and theoretical advances. Practitioners should share implementation experiences, contribute benchmark datasets, and provide feedback on framework applicability. Together, these efforts will transform the vision of AI-augmented reliability from conceptual framework into operational reality, enabling safer, faster, and more predictable software delivery for organizations of all sizes.

## Data Availability

The original contributions presented in the study are included in the article/supplementary material, further inquiries can be directed to the corresponding author.
